# Symbiotic bacteria associated with entomopathogenic nematodes showed molluscicidal activity against *Biomphalaria glabrata*, an intermediate host of *Schistosoma mansoni*

**DOI:** 10.1186/s13071-024-06605-x

**Published:** 2024-12-22

**Authors:** Jiranun Ardpairin, Chanakan Subkrasae, Abdulhakam Dumidae, Supawan Pansri, Chanatinat Homkaew, Wipanee Meesil, Tewarat Kumchantuek, Ittipon Phoungpetchara, Adler R. Dillman, Coralie Pavesi, Helge B. Bode, Sarunporn Tandhavanant, Aunchalee Thanwisai, Apichat Vitta

**Affiliations:** 1 Department of Microbiology and Parasitology, Faculty of Medical Science, Naresuan University, Phitsanulok, 65000 Thailand; 2 Department of Anatomy, Faculty of Medical Science, Naresuan University, Phitsanulok, 65000 Thailand; 3 Department of Nematology, University of California, Riverside, CA 92521 USA; 4 Max-Planck-Institut für Terrestrische Mikrobiologie Abteilung Naturstoffe in organismischen Interaktionen, Karl-von-Frisch-Str. 10, 35043 Marburg, Germany; 5 Department of Natural Products in Organismic Interactions, Max Planck Institute for Terrestrial Microbiology, 35043 Marburg, Germany; 6 Molecular Biotechnology, Department of Biosciences, Goethe University, Frankfurt, 60438 Frankfurt am Main, Germany; 7 Chemical Biology, Department of Chemistry, Philipps University Marburg, 35032 Marburg, Germany; 8 Senckenberg Gesellschaft für Naturforschung, Frankfurt am Main, Germany; 9 SYNMIKRO (Zentrum für Synthetische Mikrobiologie), 35032 Marburg, Germany; 10 Department of Microbiology and Immunology, Faculty of Tropical Medicine, Mahidol University, Bangkok, 10400 Thailand; 11 Centre of Excellence in Medical Biotechnology (CEMB), Faculty of Medical Science, Naresuan University, Phitsanulok, 65000 Thailand; 12 Centre of Excellence for Biodiversity, Faculty of Sciences, Naresuan University, Phitsanulok, 65000 Thailand

**Keywords:** Entomopathogenic nematode, Histological alteration, Schistosome, Snail, Symbiotic bacteria

## Abstract

**Background:**

*Biomphalaria glabrata* acts as the intermediate host of schistosomes that causes human schistosomiasis. Symbiotic bacteria, *Xenorhabdus* and *Photorhabdus* associated with *Steinernema* and *Heterorhabditis*, produce secondary metabolites with several biological activities. Controlling *B. glabrata* is a potential strategy to limit the transmission of schistosomiasis. The aims of this study were to identify *Xenorhabdus* and *Photorhabdus* bacteria based on *recA* sequencing and evaluate their molluscicidal activity against *B. glabrata* snail.

**Results:**

A total of 31 bacterial isolates belonging to *Xenorhabdus* (*n* = 19) and *Photorhabdus* (*n* = 12) (*X. ehlersii*, *X. stockiae*, *X. indica*, *X. griffinae*, *P. luminescens*, *P. akhurstii*, and *P. laumondii* subsp. *laumondii* were molecularly identified based on *recA* sequencing. Five isolates of bacterial extracts showed potential molluscicide, with 100% snail mortality. *P. laumondii* subsp. *laumondii* (bALN19.5_TH) showed the highest effectiveness with lethal concentration (LC) values of 54.52 µg/mL and 89.58 µg/mL for LC_50_ and LC_90_, respectively. Histopathological changes of the snail were observed in the head–foot region, which showed ruptures of the epithelium covering the foot and deformation of the muscle fiber. A hemocyte of the treated snails was observed in the digestive tubules of the digestive glands. The hermaphrodite glands of treated snails showed a reduction in the number of spermatozoa, degeneration of oocytes, and deformation and destruction in the hermaphrodite gland. In addition, liquid chromatography–tandem mass spectrometry (LC–MS/MS) of three symbiotic bacteria contained compounds such as GameXPeptide, Xenofuranone, and Rhabdopeptide.

**Conclusions:**

Five bacterial extracts showed good activity against *B. glabrata*, especially *P. laumondii* subsp. *laumondii* and *X. stockiae*, which produced virulent secondary metabolites resulting in the death of the snails. They also caused histopathological alterations in the foot, digestive glands, and hermaphrodite glands of the snails. This study suggests that extracts from these bacteria show promise as molluscicides for the control of *B. glabrata*.

**Graphical abstract:**

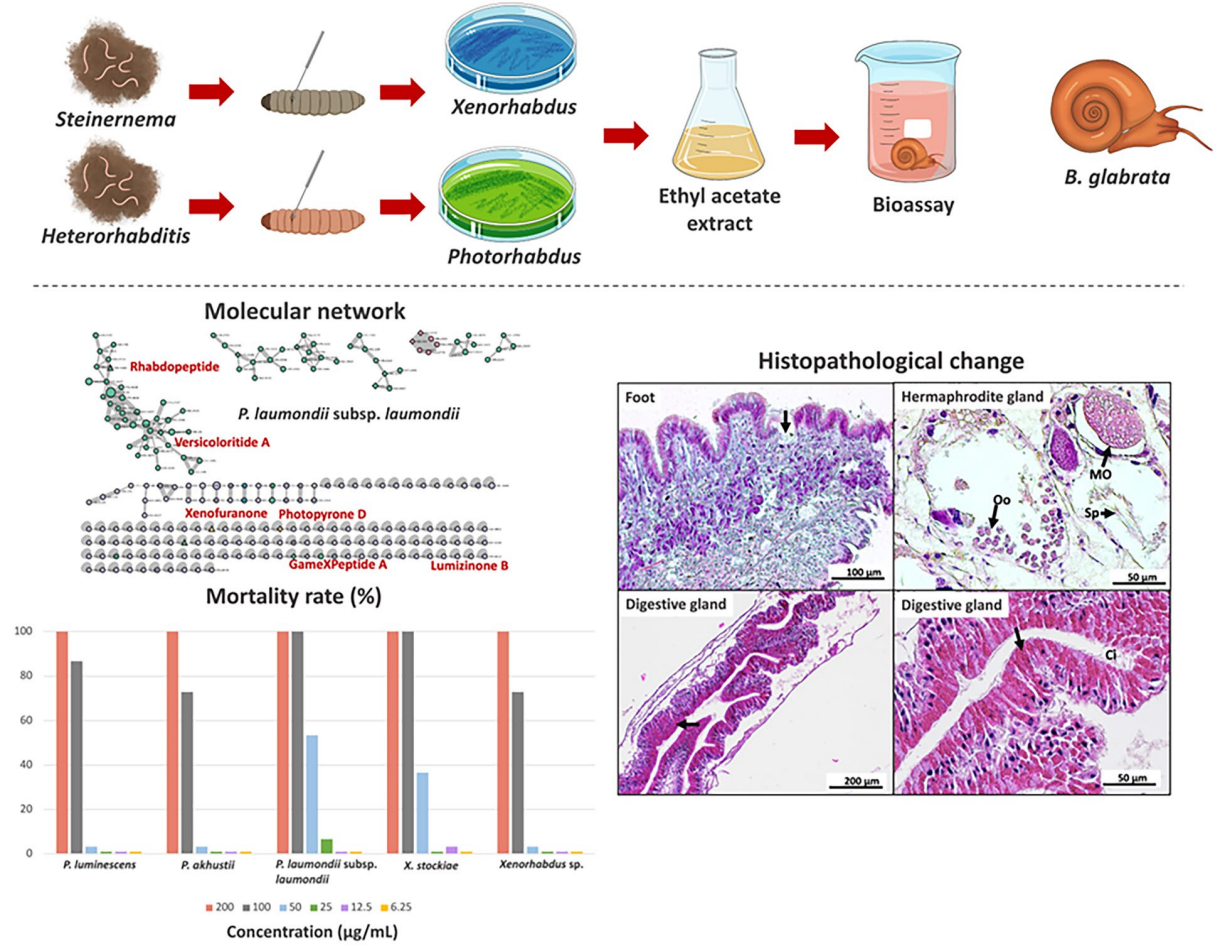

**Supplementary Information:**

The online version contains supplementary material available at 10.1186/s13071-024-06605-x.

## Background

*Biomphalaria glabrata* is an aquatic pulmonate gastropod in the family Planorbidae. This snail acts as the intermediate host of the trematode parasite, *Schistosoma mansoni*, which is the most important cause of schistosomiasis in humans. At present, over 250 million people in 78 countries are affected by this disease. Moreover, due to the COVID-19 pandemic since 2021, work to control this schistosomiasis has been reduced [1]. Chronic schistosomiasis can cause significant disability and, in some cases, can result in death [2]. The World Health Organization (WHO) recommends the use of mass drug administration (MDA) with the anthelminthic praziquantel to control schistosomiasis. However, MDA is not 100% successful. Furthermore, no licensed or commercial schistosomiasis vaccine is currently available in the market against any strain or species of schistosome infecting humans [3, 4]. Other available control approaches include environmental management, health education, and intermediate host snail control, which is the most practical approach to interrupt the schistosome life cycle [2, 5]. The molluscicide recommended by WHO is niclosamide, which is the principal compound used for controlling snail populations. There are certain limitations to using niclosamide, including limitations to the usage of potable water immediately after application and temporary high toxicity to other aquatic animals including fish, amphibians, and humans [6]. Also, the high cost and appearance of snails resistant to niclosamide has limited its usefulness in controlling the snails [7, 8]. The development of novel molluscicides of synthetic or biological origin is an important approach. The use of plant-based molluscicides (extracts and compounds isolated from plants) is one of the most effective methods to reduce snail populations [9–11]. Biological control agents such as entomopathogenic nematodes (EPNs) of the genera *Steinernema* and *Heterorhabditis* have shown great potential to control insect pests as well as the intermediate host snail [12–14].

Symbiotic bacteria, *Xenorhabdus* and *Photorhabdus*, are associated with EPNs in the genus *Steinernema* and *Heterorhabditis*, respectively [15]. The infective juvenile stage (IJs) of EPNs contain symbiotic bacteria in their intestines. When the EPNs penetrate the insect host by entering through an orifice such as the mouth, anus, or spiracles, or by passing directly through the cuticle, they release their bacteria, which can produce several secondary metabolites that act as cytotoxic, antibacterial, antifungal, antiparasite, and insecticidal agents in the blood system of the insect [16, 17]. The IJs of EPNs kill the insect within 24–48 h [18–20]. Moreover, previous studies have demonstrated the pathogenicity of EPNs to gastropod snails. For example, exposure to *Heterorhabditis indica* LPP1 caused 55% mortality in *Bradybaena similaris*, with a significant reduction (*P* < 0.05) in the glucose content of the hemolymph of the exposed snails, indicating that infection by *H. indica* [21, 22]. A subsequent study in 2017 evaluated susceptibility of *Lymnaea columella* to *H. baujardi*, with mortality rate of 66% and demonstrated the strong pathogenic potential of *H. baujardi* to control this snail [13, 23]. Recently, the susceptibility of *B. glabrata* to the *H. bacteriophora* isolate HP88 was reported. The infection induces breakdown of carbohydrate homeostasis in *B. glabrata* but did not show lethality in the population of infected snails [14]. While previous studies have demonstrated the pathogenicity of EPNs to the snails, to our knowledge, no previous study has been conducted with the symbiotic bacteria, *Photorhabdus* and *Xenorhabdus* associated with EPNs, to control the snails.

*Photorhabdus* and *Xenorhabdus* are motile Gram-negative bacteria belonging to the family Enterobacteriaceae [24]. These bacteria have been used as biocontrols for a variety of insect pests, insect vectors, and pathogens [25, 26]. Approximately 24 species of *Xenorhabdus* and five species of *Photorhabdus* have been described to have insecticidal activity [27]. Our previous study reported the isolation and identification of EPNs isolated from agricultural areas in Thailand [28]. This study continues our work on the isolation and identification of symbiotic bacteria based on sequencing of the recombinase A (*recA*) gene. We hypothesized that symbiotic bacteria might be a potential molluscicide for controlling *B. glabrata*. In addition, liquid chromatography–tandem mass spectrometry (LC–MS/MS) analysis together with molecular networking of symbiosis bacteria extracts was performed to elucidate the secondary metabolites produced by the selected symbiotic bacteria. Therefore, this research aimed to identify symbiotic bacteria and evaluate their molluscicidal activity against *B. glabrata*.

## Methods

### Isolation and identification of *Photorhabdus* and *Xenorhabdus*

The methods for soil collection and EPN isolation were previously described by Ardpairin et al. [28]. Five isolates of EPNs including *Steinernema. surkhetense* (eAST17.4_TH), *S. lamjungense* (eALN10.5_TH), *H. bacteriophora* (eALN19.5_TH), *H. indica* (eACM14.2_TH), and *H. indica* (eAPY3.5_TH) were used for infecting waxworms (*Galleria mellonella*). A few drops of suspension of 300–500 IJs of each EPN isolate were transferred to a small Petri dish containing five waxworm larvae. Subsequently, the Petri dishes were sealed with Parafilm and incubated at room temperature for 2–4 days. The waxworm larvae were observed daily for 2–3 days. Symbiotic bacteria, *Xenorhabdus* and *Photorhabdus*, were isolated from the dead waxworms infected with the IJs of EPNs. The dead waxworms were washed with sterilized water, and were surface sterilized by dipping into absolute ethanol. Then, they were air dried in a sterile Petri dish. The third segment from the head of waxworms was dissected using sterile forceps. Hemolymph of the waxworms was touched by a sterile loop and subsequently streaked on nutrient agar supplemented with bromothymol blue and triphenyl-2,3,5-tetrazolium chloride (NBTA). The NBTA plates were sealed with Parafilm and then incubated in the dark at room temperature (25–28 °C). After 4 days of incubation, *Xenorhabdus* and *Photorhabdus* colonies were observed as blue and green on NBTA, respectively [29].

To identify *Photorhabdus* and *Xenorhabdus* bacteria, PCR and DNA sequencing based on a partial *recA* gene was performed, as previously described [30] for all symbiotic bacteria. This *recA* gene is a conserved gene across many bacteria with some variabiations that make it effective for distinguishing between species that are closely related. In brief, a single colony of each isolate was inoculated in 5 mL Luria–Bertani (LB) broth, then incubated and shaking at 180 rpm for 18–24 h. The genomic DNA of the symbiotic bacteria was extracted using the Genomic DNA mini kit (blood/culture cell; Geneaid Biotech Ltd., New Taipei, Taiwan). The genomic DNA was stored at −20 °C for further amplification by PCR. The partial region of the *recA* gene (890 bp) was amplified using the following primers: recA_F (59-GCTATTGATGAAAATAAACA-39) and recA_R (59-RATTTTRTCWCCRTTRTAGCT-39) [31]. The total volume of PCR reagents (30 μL) containing 1.5–μL genomic DNA, 15 μL of EconoTaq PLUS Master Mix, 1.5 μL of each primer (1 μM), and 10.5 μL of distilled water. All PCR reaction tubes were placed into an Applied Biosystems thermal cycler (Carlsbad, CA). The PCR cycling conditions were predenaturation at 94 °C for 5 min, followed by 30 cycles of denaturation at 94 °C for 1 min, annealing at 45 °C for 45 s, extension at 72 °C for 2 min, and a final extension at 72 °C for 7 min. PCR products were visualized on a 1.2% agarose gel stained with ethidium bromide and purified using a PCR clean-up Gel Extraction Kit (Macherey–Nagel, Düren, Germany). PCR products were sequenced from forward and reverse directions by Macrogen Inc. (South Korea). All nucleotide sequences were checked and edited using SeqManII (DNASTAR Inc., Madison, WI, USA) and identified by comparing edited sequences with known sequences in GenBank using a BLASTN search (http://www.ncbi.nlm.nih.gov/blast/Blast.cgi). The similarity of the sequences between our sequences and known sequences with ≥ 97.00 was considered the same species.

### Phylogenetic analysis

The nucleotide sequences from this study were aligned with known species and subspecies of *Xenorhabdus* and *Photorhabdus* using ClustalW in the MEGA program version 7.0 [32]. Maximum likelihood trees were constructed using the IQ-Tree program v2.3.6 with 1000 ultrafast bootstraps. The best-fit model selected by the IQ-Tree program for *Xenorhabdus* was TIM3e + I + G4 and TNe + I + R2 for *Photorhabdus* [33].

### Culture of *Biomphalaria glabrata*

*B. glabrata* snails were obtained from the Department of Helminthology, Faculty of Tropical Medicine, Mahidol university, Bangkok, Thailand. The snails were maintained and bred in 120 L aquarium filled with dechlorinated water, temperature of 25 ± 3 °C, under 12 h:12 h light:dark cycles and fed with fresh lettuce in the Department of Microbiology and Parasitology, Faculty of Medical Science, Naresuan University.

### Preparation of symbiotic bacteria extract

The selected *Xenorhabdus* and *Photorhabdus* isolates were subcultured on NBTA plates, incubated at room temperature in the dark for 4 days. A single colony of each isolate was cultured in 500 mL tryptic soy broth (TSB) at 28 °C, 180 rpm for 72 h using a shaking incubator. To extract the crude bioactive compound, 1000 mL of ethyl acetate was added to the 500 mL bacteria culture and mixed well. Subsequently, the mixture flask was placed at room temperature for at least 24 h. A rotary vacuum evaporator (Buchi, Flawil, Switzerland) was used to evaporate the ethyl acetate from the mixture to concentrate all the bacterial extracts. This process was performed three times to maximize the crude extract. The condensed bacteria extract of each isolate was weighed and dissolved in DMSO to obtain different concentrations of 20, 10, 5, 2.5, 1.25, and 0.625 mg/mL. These stock solutions were stored at −20 °C. Before being used, each stock solution of bacterial extract was diluted in dechlorinated water to obtain the final different concentration (200, 100, 50, 25, 12.5, and 6.25 µg/mL).

### Evaluation of symbiotic bacteria extract against snails

A bioassay of the molluscicidal activity against the snail was determined according to the guidelines for laboratory and field testing of molluscicides for control of schistosomiasis [34]. There were six final concentrations of each symbiotic bacterial extract (200, 100, 50, 25, 12.5, and 6.25 µg/mL), and three control groups with two negative controls (dechlorinated water and 1% DMSO) and a positive control (1.0 µg/mL niclosamide). Ten *B. glabrata* snails with shell diameters of 10–14 mm [10] were set up for each concentration. The snails were exposed to 500 mL of dechlorinated water mixed with each bacterial extract in 600 mL beakers for 24 h. Subsequently, the snails were washed with dechlorinated water and then placed in new beakers and fed with fresh lettuce. Monitoring the mortality of snails was performed at 24, 48, and 72 h. Snails that remained completely within their shells and showed no movement were suspected to be dead. For confirmation of death, the soft tissue of the snail was stimulated with a needle to determine whether there was a contractile response and heart movements were observed under a stereo microscope. The entire experiment was conducted in triplicate on different days. Statistical analysis of mortality rate was compared using ANOVA with Bonferroni’s post hoc for multiple comparisons. The analyses were performed in GraphPad Prism 10.3.1. The difference was considered statistically significant when the *P*-value was less than 0.05. The LC_50_ and LC_90_ with 95% confidence interval were calculated by regression analysis in Excel software.

### Histopathological study of *B. glabrata*

The snails were tested with the LC_50_ of all selected bacterial extracts to investigate histopathological changes. After 3, 6, 12, and 24 h of exposure, snails were transferred to Davidson’s rfixative for 72 h and shucked in 14% EDTA to decalcify the shell. Snail tissues were washed and dehydrated in a graded ethanol series. Dehydrated snails were cleared with xylene solution, then each snail tissue was embedded in paraffin. Serial sections were cut at 5 μm thickness using a rotary microtome (Leica RM2235, Wetzlar, Germany). The sections that were mounted on glass slide were deparaffinized with xylene then stained with hematoxylin for 5 min, rinsed with tap water, followed by eosin for 3 min. The slides with stained snail tissue were mounted using Permount. The presence of histological alterations was observed under a light microscope (Olympus BX51, Tokyo, Japan). Images were captured by digital camera.

### LC–MS/MS analysis of symbiosis bacteria extracts

The methanol crude extract of three symbiotic bacteria was prepared for analysis of the secondary metabolites. LC–MS/MS analysis was conducted using a Dionex Ultimate 3000 system coupled to an AmaZonX mass spectrometer (Bruker Daltonics). The data were obtained on a timsTOF fleX MALDI-2 with a column Acquity UPLC BEH C18 2.1 × 50 mm (solvent A = H_2_O + 0.1% formic acid, solvent B = acetonitrile + 0.1% formic acid), gradient ranging from 5% to 95% over 16 min, with a flow rate of 0.4 mL/min. A molecular network was generated using the MS cluster online workflow available at GNPS (http://gnps.ucsd.edu/ProteoSAFe/static/gnps-splash.jsp) along with manual dereplication with NPAtlas and LOTUS based on the molecular formula. Cytoscape v3.10.2 was utilized to visually represent the data as a network of nodes and edges.

## Results

### Identification of symbiotic bacteria

A total of 31 isolates of symbiotic bacteria were identified as 19 and 12 isolates of *Xenorhabdus* (GenBank accession nos OR506420–OR506439) and *Photorhabdus* (GenBank accession nos OR506440–OR506451), respectively. BLASTN search of *recA* sequences revealed that six isolates of *Xenorhabdus* showed sequence similarity to *X. stockiae* strain CS33 at above 99% identity. Three *Xenorhabdus* isolates were closely related to *X. stockiae* strain TH01, and another one isolate showed sequence similarity to *X. stockiae* strain 858516. The other two *Xenorhabdus* isolates in this study showed sequence similarity to *X. griffiniae* strain ID10 with 99.53% and 99.69% identity. One isolate of *Xenorhabdus* was closely related to *X. indica* strain SabOM with 96.71% identity. The remaining six isolates of *Xenorhabdus* showed sequence similarity ranging from 95.77% to 96.08% to *X. ehlersii* strain DSM16337. Twelve isolates of *Photorhabdus* were identified as *P. akhurstii* (five isolates) and *P. laumondii* subsp. *laumondii* (three isolates). The other four isolates were closely related to *P. luminescens* strain C8404. The sequences of *Photorhabdus* showed sequence similarity ranged from 98.50% to 100.00%.

### Phylogenic analysis of *Xenorhabdus* and *Photorhabdus*

**Fig. 1:**
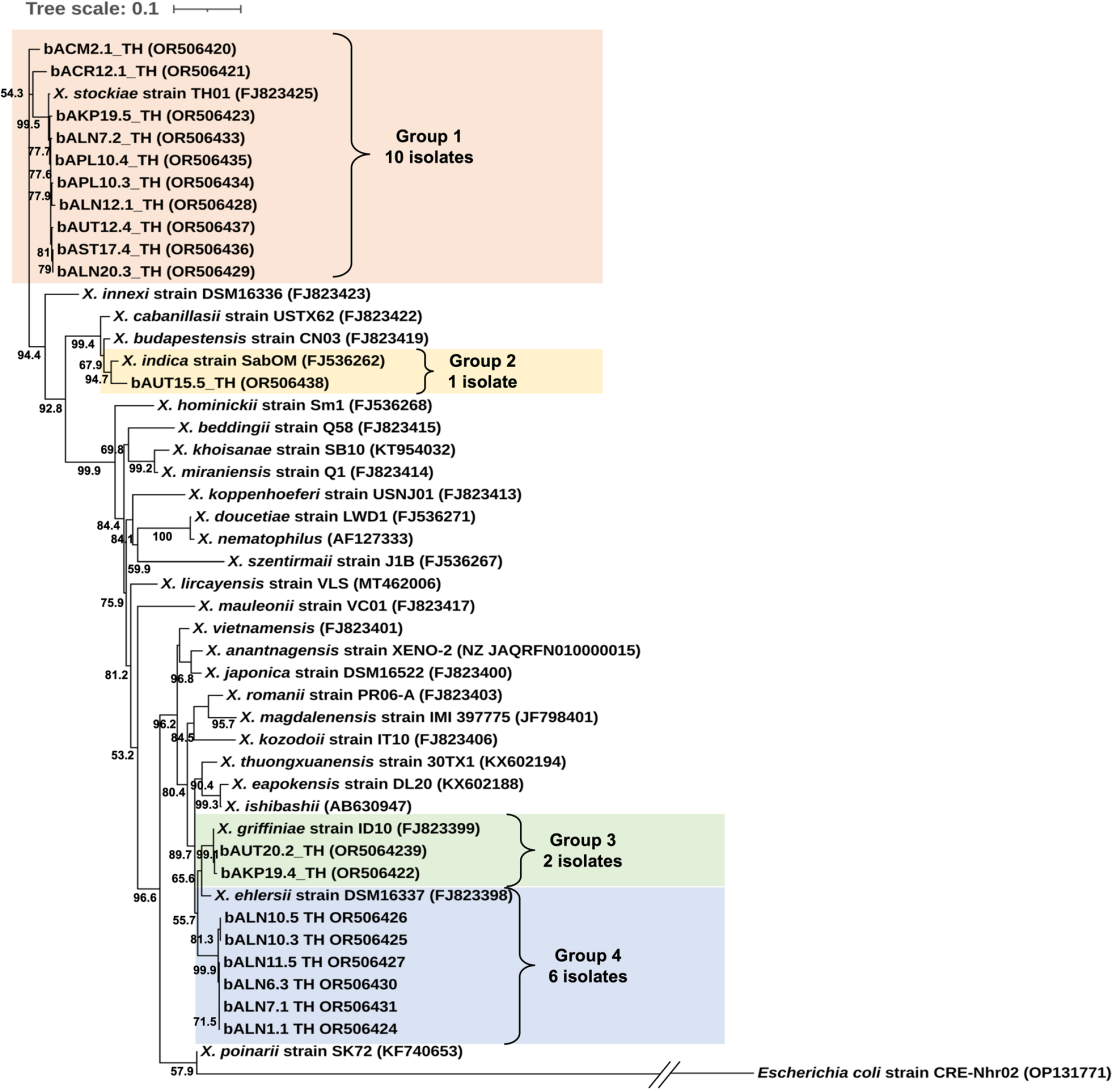
The phylogeny of *Xenorhabdus* (maximum likelihood method, bootstrap = 1000) based on 638 nucleotides of a partial region of the *recA* gene from 19 *Xenorhabdus* isolates in the present study together with several *Xenorhabdus* species retrieved from the NCBI database. *Escherichia coli* was used as the out group

**Fig. 2:**
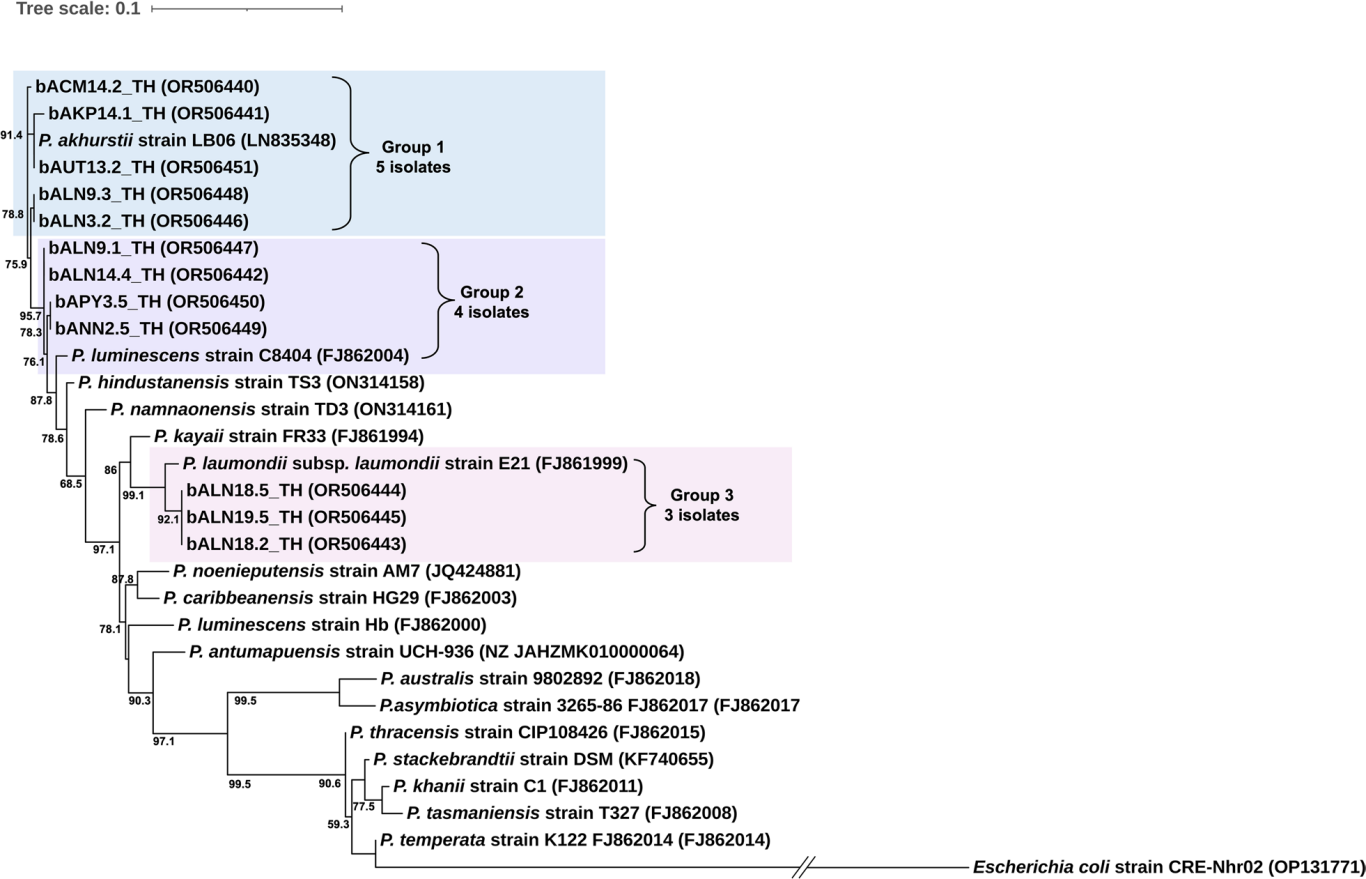
The phylogeny of *Photorhabdus* (maximum likelihood method, bootstrap = 1000) based on 588 nucleotides of a partial region of the *recA* gene from 12 *Photorhabdus* isolates in the present study together with several *Photorhabdus* species retrieved from the NCBI database. *Escherichia coli* was used as the out group

The phylogenetic tree based on maximum likelihood analyses revealed four groups of *Xenorhabdus*. Group 1 contained ten isolates of *Xenorhabdus* in this study, which were clustered with *X. stockiae* strain TH01 (accession no. FJ823425). Group 2 contained only one isolate, which was grouped with *X. indica* strain SabOM (accession no. FJ536262). Group 3 contained two isolates of *Xenorhabdus* in this study together with *X. griffiniae* strain ID10 (accession no. FJ823399) received from the NCBI database. Group 4 contained six isolates of *Xenorhabdus* in this study, which were clustered with *X. ehlersii* strain DSM16337 (accession no. FJ823398) (Fig. 1). Twelve isolates of *Photorhabdus* in this study were divided into three groups. Group 1 contained five isolates of *Photorhabdus* and the referenced *P. akhurstii* strain LB06 (accession no. LN835348). Group 2 contained four isolates of *Photorhabdus* in the present study and *P. luminescens* strain C8404 (accession no. FJ862004). Group 3 was composed of three isolates of *Photorhabdus* in this study, which were closely related to *P. laumondii* subsp. *laumondii* strain E21 (accession no. FJ861999) (Fig. 2).

### Molluscicidal activity of symbiotic bacteria against *B. glabrata*

**Fig. 3:**
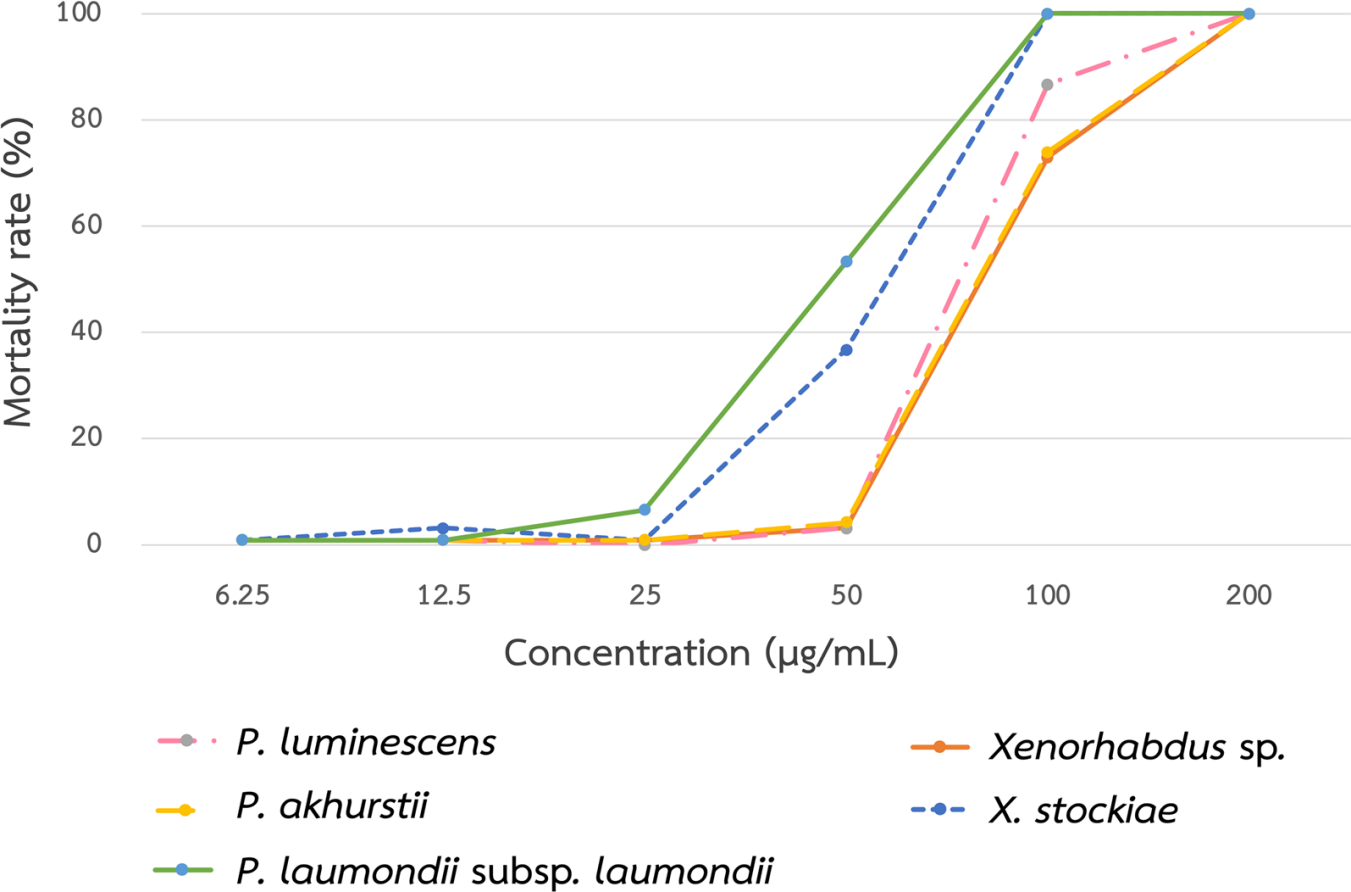
Mortality rates of *Biomphalaria glabrata* at different concentrations of bacterial extract

Adult *B. glabrata* were exposed to crude extracts of five symbiotic bacteria: *X. stockiae* (bAST17.4_TH), *Xenorhabdus* sp. (bALN10.5_TH), *P. laumondii* subsp. *laumondii* (bALN19.5_TH), *P. akhurstii* (bACM14.2_TH), and *P. luminescens* (bAPY3.5_TH). The results showed significant differences from the negative control for all extracts tested at concentrations 100 and 200 µg/mL (*P* < 0.001) (Supplementary Table S1). All bacterial isolates showed good potential in controlling *B. glabrata* at concentrations of 200 µg/mL. Snail mortality reached 100% within 72 h. The positive control was exposed to 1% niclosamide causing 100% mortality at 24 h. Two from the 300 snails of the negative controls, treated with 1% DMSO and DW, died. One hundred percent mortality was also observed from two isolates of symbiotic bacteria (bAST17.4_TH and bALN19.5_TH) at a concentration of 100 µg/mL. The extract from *P. laumondii* subsp. *laumondii* (bALN19.5_TH) showed the highest effectiveness for killing *B. glabrata* after 24 h exposure, with LC values of 56.58 µg/mL and 92.24 µg/mL for LC_50_ and LC_90_, respectively. At 72 h of exposure, the LC values were 54.52 (52.47–56.61) µg/mL and 89.58 (87.35–91.84) µg/mL for LC_50_ and LC_90_, respectively (Table 1). The extract from *X. stockiae* (bAST17.4_TH) also showed high effectiveness, with LC values at 72 h of 58.57 (57.18–60.05) µg/mL and 94.61 (92.54–96.85) µg/mL for LC_50_ and LC_90_, respectively (Table 1). The mortality rate of other isolates ranged between 73% and 86.7% at 100 µg/mL after 72 h. Testing 50 µg/mL showed low activity against *B. glabrata* snail in all bacterial isolates (Fig. 3).

**Table 1:** Lethal concentrations of bacterial extract for killing *Biomphalaria glabrata* during 72 h

Symbiotic bacteria (code)	Lethal concentration and 95% CI^*^ (µg/mL)	
	LC_50_	LC_90_
*P. luminescens* (bAPY3.5_TH)	96.40 (94.46–98.38)	163.56 (161.31–165.87)
*P. akhurstii* (bACM14.2_TH)	101.27 (98.83–103.48)	170.43 (167.63–172.90)
*P. laumondii* subsp. *laumondii* (bALN19.5_TH)	54.52 (52.47–56.61)	89.58 (87.35–91.84)
*Xenorhabdus* sp. (bALN10.5_TH)	101.27 (99.92–102.37)	170.43 (168.57–171.96)
*X. stockiae* (bAST17.4_TH)	58.57 (57.18–60.05)	94.61 (92.54–96.85)

^a^95% Confidence interval

### Histopathological change of *B. glabrata*

Pathological changes were not observed in the head–foot region, digestive glands, or hermaphrodite glands of snails in the negative control group. The histopathological change of snails was observed after 3 h after exposure to niclosamide and *P. laumondii* subsp. *laumondii* (bALN19.5_TH). For another four bacterial extracts, histological alterations were observed after 12 h exposure.

**Fig. 4:**
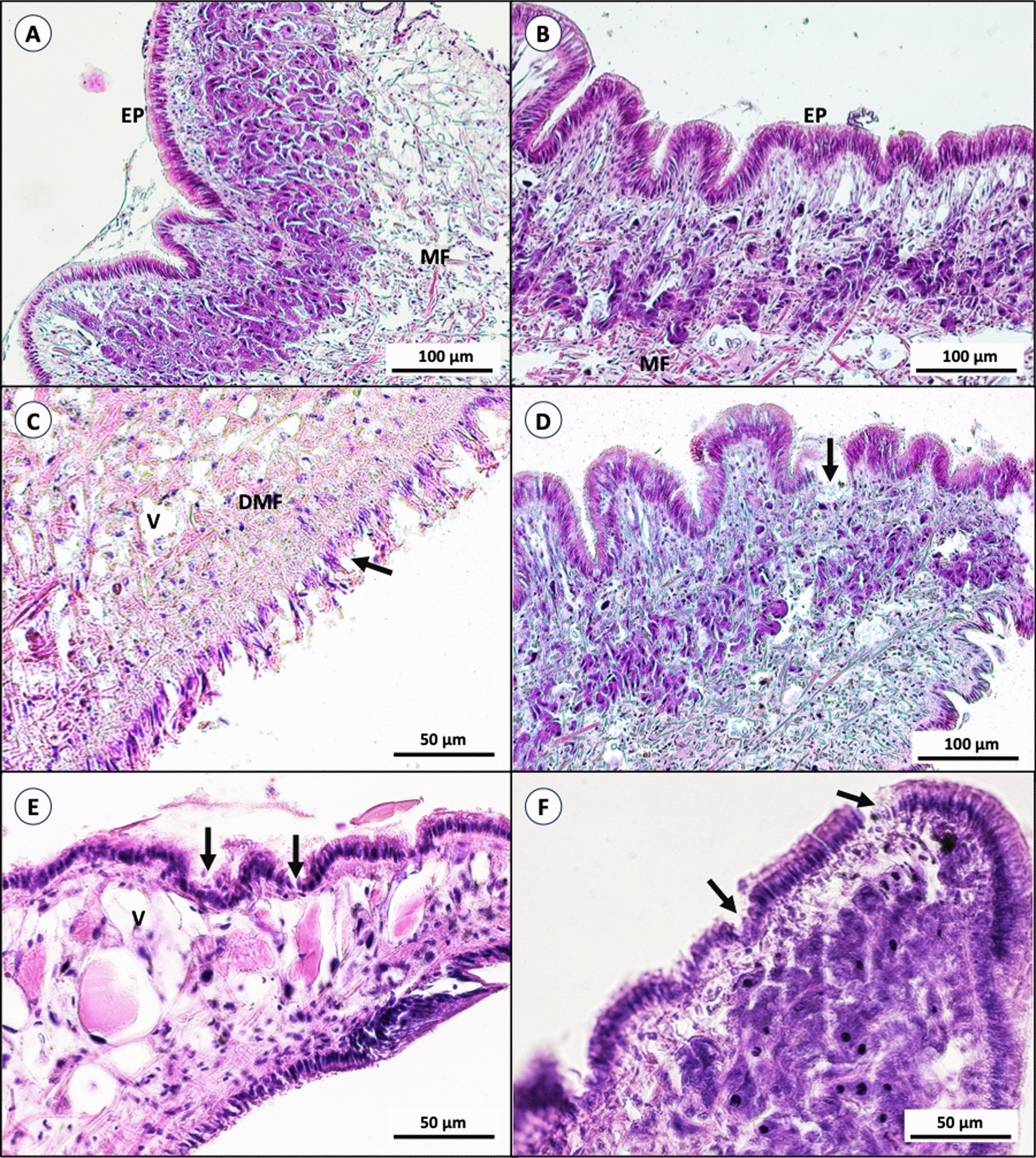
Histological section of *B. glabrata* head–foot region (Hematoxylin & Eosin stained); snails in the negative control, exposed to dechlorinated water and DMSO showing the sole of the foot covered with regular columnar epithelium (EP) (**A**, **B**). Snails treated with niclosamide showing a ruptured epithelial layer (arrow) and deformed muscle fiber (DMF) (**C**). Snails treated with LC_50_ of bacterial extract showing a ruptured epithelial layer (arrow) and empty spaces (vacuoles) (V) in the connective tissue layer (**D**–**F**)

Head–foot region: The foot of the snails is covered with a columnar epithelial layer, with the connective tissue layer containing the mucous gland beneath. The foot is the first target of toxic activity. After exposure to bacterial extracts and niclosamide, the columnar epithelia of the outer layer were ruptured, and the muscle fibers beneath the epithelial layer were destroyed and deformed. Empty spaces (vacuoles) were observed in the connective tissue. Moreover, there was dark-brown pigment and necrosis of the gland within connective tissue (Fig. 4).

**Fig. 5:**
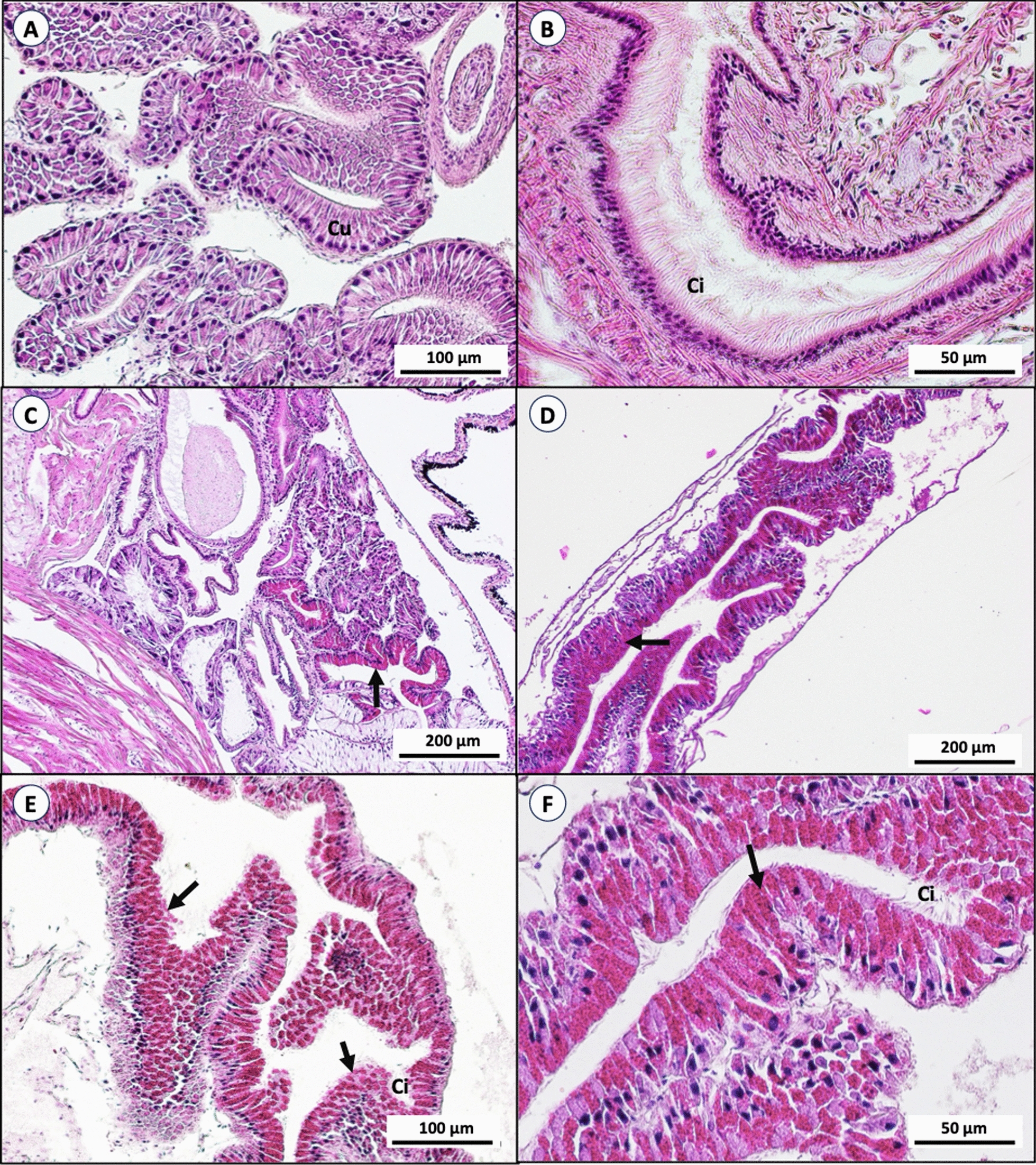
Histological sections of *B. glabrata* digestive glands (Hematoxylin & Eosin stained); snails in the negative control with normal simple columnar cells (Cu) of digestive glands and cilia (Ci) covering the digestive tract (**A**, **B**). Snails treated with niclosamide and bacterial extract present with hemocytes (arrow) in the tissue (**C**–**F**)

Digestive glands: The digestive gland of snails is lined with simple digestive columnar cells, which are cylindrical in character and have basal nuclei. The tracts are covered with ciliated cells. There was no damage to cells or tissue, and no hemocytes were present. The presence of hemocytes was observed at the columnar epithelial layer in the digestive gland after being treated with bacterial extracts and niclosamide. A decrease in ciliated cells was also observed. The morphology of cells and nuclei showed no differentiation after treatment (Fig. 5).

**Fig. 6:**
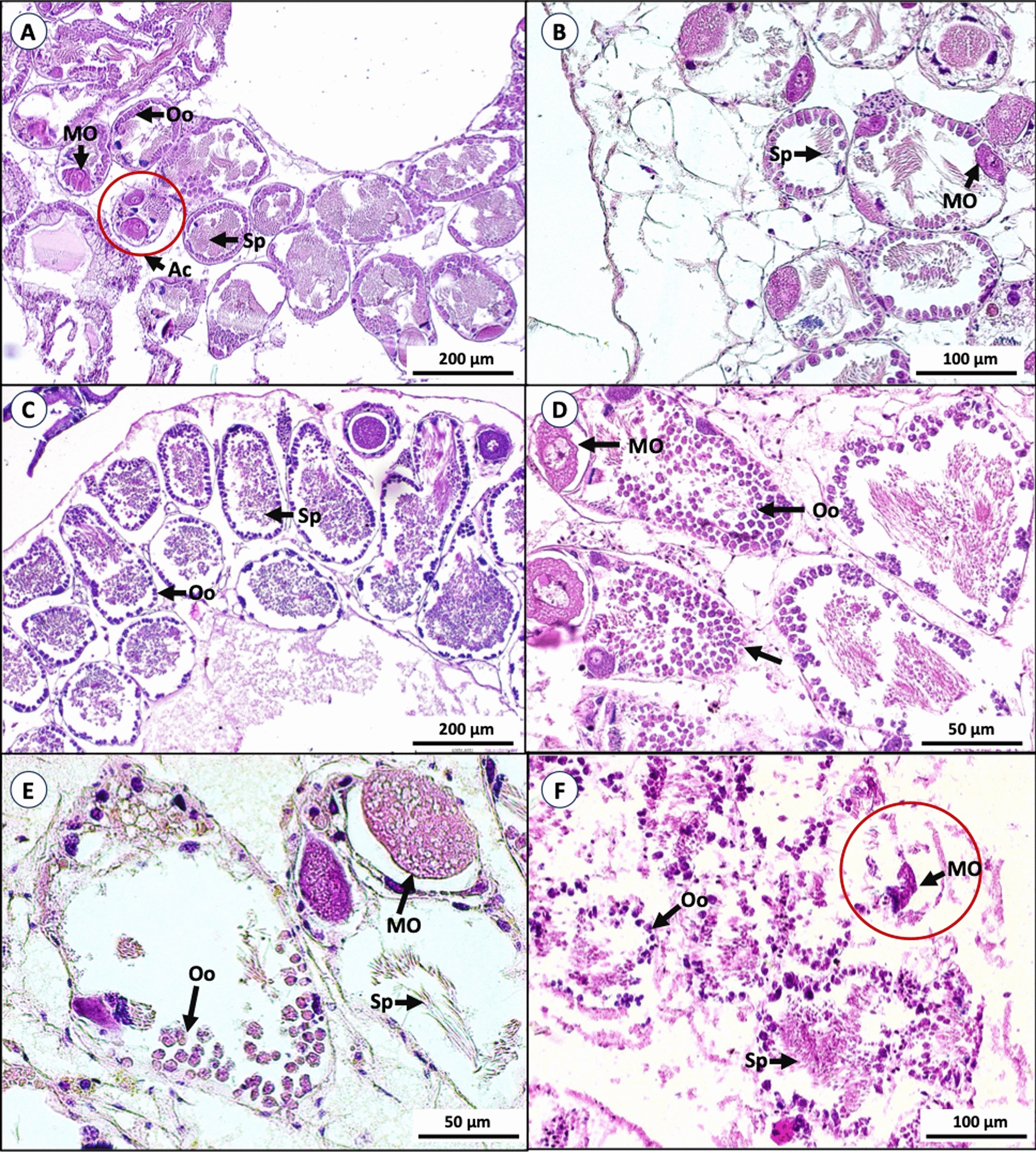
Histological section of *B. glabrata* hermaphrodite glands (Hematoxylin & Eosin stained); snails in the negative control with normal acini (Ac) containing plenty of spermatozoa (Sp), some oocytes (Oo), and mature ovum (MO) (**A**). Snails treated with niclosamide showed a decreased number of spermatozoa and degeneration of oocytes (**B**). Snails treated with bacterial extract showed mild (**C**), moderate (**D**), and severe effects (**E**, **F**), causing deformation and destruction in hermaphrodite glands

Hermaphrodite glands: The hermaphrodite gland of *B. glabrata* consists of male (spermatocytes and sperms) and female (oocytes and mature ova) reproductive gametes in the vesicles known as acini. Each acinus was separated by thin vascular connective tissue. Snails treated with bacterial extracts and niclosamide had a decreased number of spermatozoa and the oocytes inside the acini were degenerated when compared to control snails. The vascular connective tissue between acini was deformed. Sever necrotic changes and degeneration of acini were also observed (Fig. 6).

### Secondary metabolites in *Xenorhabdus* and *Photorhabdus* bacteria

**Fig. 7:**
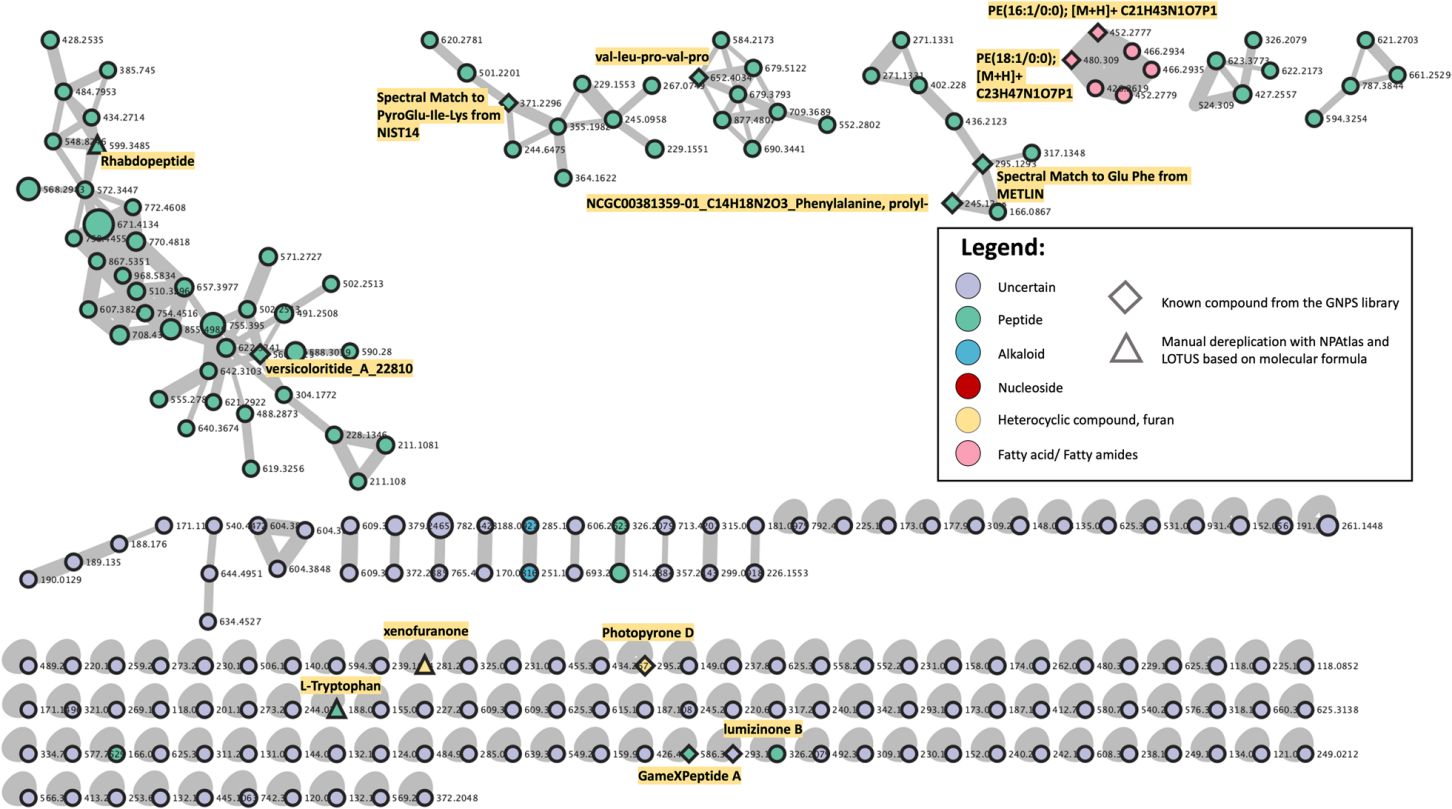
Network analysis of *Photorhabdus laumondii* subsp. *laumondii* (bALN19.5_TH) showing 223 nodes connected in networks with at least one neighbor and a single node. Thirteen known natural product compounds were highlighted in the network

Three bacterial isolates were submitted to liquid chromatography–tandem mass spectrometry (LC–MS/MS) to identify the secondary metabolites produced from each isolate. *Xenorhabdus stockiae* (bAST17.4_TH) had a greater number of single nodes (355), compared to *P. luminescens* (bAPY3.5_TH) (244) and *P. laumondii* subsp. *laumondii* (bALN19.5_TH) (223) (Supplementary Table S2). These metabolites were identified based on previous studies using the GNPS, NPAtlas, and LOTUS databases. All isolates produced GameXPeptide. *Photorhabdus laumondii* subsp. *laumondii* (bALN19.5_TH) produced GameXPeptide A (Fig. 7), while *P. luminescens* (bAPY3.5_TH) and *X. stockiae* (bAST17.4_TH) produced both GameXPeptide A and C (Supplementary Figs. S1, S2.). Prolylphenylalanine was also found in these three isolates. Rhabdopeptide and Xenofuranone were produced by *P. laumondii* subsp. *laumondii* (bALN19.5_TH) and *X. stockiae* (bAST17.4_TH). Furthermore, *P. laumondii* subsp. *laumondii* (bALN19.5_TH) was found to produce Versicoloritide A, Photopyrone D, and Lumizinone B (Fig. 7). However, most of molecules produced by these bacteria are still unknown.

## Discussion

In this study, *Photorhabdus* and *Xenorhabdus* were isolated from *Heterorhabditis* and *Steinernema* isolated from soil samples in agricultural areas in Thailand [28]. Phylogenetic analysis of 31 isolates of *Photorhabdus* and *Xenorhabdus* identified sequences closely related to *P. luminescens*, *P. akhurstii*, *P. laumondii* subsp. *laumondii*, *X. ehlersii*, *X. stockiae*, *X. indica*, and *X. griffinae* [35, 36]. The most abundant *Xenorhabdus* species found was *X. stockiae*, while *P. luminescens* and *P. akhurstii* were highly represented among the genus *Photorhabdus*. This result is consistent with the results of previous studies in Thailand, showing that *X. stockiae* and *P. akhurstii* were the most common species found in Thailand. In addition, all of bacteria found in this study were in line with the results of previous studies in Thailand [29, 30, 37–42]. Most *X. stockiae* in this study were isolated from *Steinernema surkhetense* and one isolate was from *S. siamkayai*. *X. stockiae* was previously reported with *S. siamkayai* in Thailand in 2006 [43]. Other species of EPNs that have been reported to be associated with *X. stockiae* are *Steinernema. surkhetense*, *S. huense*, and *S. websteri* [29, 37–40, 42]. Two isolates of *X. griffiniae* were isolated from *Steinernema. surkhetense*. This species was first isolated from *S. hermaphroditum* in Indonesia [43]. Recently, Subkrasae et al. (2022) [30] also reported an association between *X. griffiniae* and *Steinernema adamsi* [44]. An isolate of *X. indica* was found with an unknown species of *Steinernema*. *X. ehlersii* was shown to be associated with *S. lamjungense*, which is interesting, considering that previous studies have reported *X. ehlersii* to be associated with *S. serratum* [45], *S. scarabaei* [38], and *S. longicaudum* [43]. Three species of *Photorhabdus* were isolated from *H. indica* and *H. bacteriophora*. *H. indica* is a host for *P. luminescens* and *P. akhustii* in this study. These results are similar to previous reports from Thailand [29, 30, 46] and reports from Australia, Cuba, the Dominican Republic, Israel, Jamaica, and Puerto Rico [25, 47]. *P. laumondii* subsp. *laumondii* was previously reported in association with *H. bacteriophora* [29, 36, 47]. In the present study, all *P. laumondii* subsp. *laumondii* were also found to be associated with *H. bacteriophora*. The new associations between EPNs and symbiotic bacteria observed in this study include *Steinernema. surkhetense*—*X. griffiniae* and *S. lamjungense*—*X. ehlersii*.

Strategies that search for alternative molluscicides to decrease snail populations and interrupt the schistosome life cycle have been provided by the WHO’s road map and planned for 2030 [2]. Herein, we reported the first evaluation of symbiotic bacteria, *Photorhabdus* and *Xenorhabdus* against *B. glabrata* snail. All ethyl acetate extracts of symbiotic bacteria showed good molluscicidal activity against *B. glabrata*. However, according to the WHO criterion, the crude extract obtained from plants is considered active against freshwater snails at concentrations up to 100 μg/mL, which causes 70–100% mortality of snails at the 24 h exposure time [48]. Two species of bacteria tested (*P. laumondii* subsp. *laumondii* (bALN19.5_TH) and *X. stockiae* (bAST17.4_TH)) cause 100% mortality at a concentration of 100 µg/mL. The most effective bacterium in this study was *P. laumondii* subsp. *laumondii* (bALN19.5_TH), which showed LC values of 56.58 µg/mL and 92.24 µg/mL for LC_50_ and LC_90_, respectively. In this study, *P. laumondii* subsp. *laumondii* (bALN19.5_TH) was isolated from the nematode, *H. bacteriophora*. Recently, the susceptibility of *B. glabrata* to IJs of *H. bacteriophora* (isolate HP88) was reported under laboratory conditions. The results indicated that the infection induces breakdown of carbohydrate homeostasis in *B. glabrata* but did not cause lethality in the population of infected snails [14]. Recently, Dumidae et al. reported that ethyl acetate extracts from *P. laumondii* subsp. *laumondii* and *P. akhurstii* had an effect on the epithelial cells and foot muscle of *Indoplanorbis exustus* and *Radix rubiginosa*, respectively [49]. Considering other substances that have been evaluated to control *B. glabrata*, crude extracts and ethyl acetate fractions of *Manilkara subsericea* leaves induced 80 ± 4.13% and 86.66 ± 4.59% mortality of adult snails at concentrations of 250 µg/mL after 96 h [50]. Curcumin caused 53% mortality in adult snails after exposure to 100 µg/mL [11]. The essential oil extracted from *Eryngium triquetrum* caused 100% mortality in *B. glabrata* and the LC_50_ and LC_90_values for the snails were 0.61 and 1.02 µg/mL, respectively, at 24 h exposure [51]. The usnic acid (UA), isolated and purified from *Cladonia substellata* caused 87% and 100% mortality against *B. glabrata* at concentrations of 3 µg/mL and 4 µg/mL, respectively [10]. A possible mechanism of UA’s action is that it diffuses through the cell membrane and affects the electron transport chain, which can lead to biochemical and physiological changes to cell homeostasis and result in cell apoptosis [52]. Most of the purified extracts showed high efficacy against snails at low concentrations. However, the bacterial extracts in this study showed molluscicidal activities at concentrations lower than the limits established by the WHO. Therefore, the extracts from *Xenorhabdus* and *Photorhabdus* bacteria showed promising results as alternative molluscicides for the control of *B. glabrata*. The toxicity to other organisms in the environment was not evaluated in this study; however, *Xenorhabdus* and *Photorhabdus* are well known to be pathogenic to insects and not mammals. However, *Photorhabdus asymbiotica* can also infect humans [53].

Ethyl acetate extract of *Xenorhabdus* and *Photorhabdus* affected cells in the snails. The first target of toxic activity was the head–foot region. After being treated with the LC_50_ of bacterial extract, the foot of snails showed histological changes, with the destruction of the epithelial layer and presenting dark brown pigment. These results are similar to a previous report of Abdel-Rahman, 2020. The land snail *Monacha* sp. was exposed to essential plant oils and the foot of the snail showed slight shrinkage and rupture of muscular tissue, migration and precipitation of pigment cells, and slight distortion of epithelial covering [54]. Similar results were previously reported, in that the foot of *M. obstructa* snails treated with the LC_50_ of Jatropha showed rupture of the epithelium covering the foot [55]. In addition, the foot of *B. siamensis goniomphalos* snails treated with camellia and mangosteen extracts showed disruption of columnar muscle fibers and gaps between epithelial cells and connective tissue. Moreover, protein and pigment cells were found to be concentrated [56]. LC–MS/MS analysis of the three isolates that are most effective in controlling snails contained known compounds such as GameXPeptide, Rhabdopeptide, Xenofuranone, and Photopyrone. All three isolates produced GameXPeptides, which are a class of cyclic pentapeptides initially believed to be widespread in *Photorhabdus* and *Xenorhabdus* and significant for these bacteria [57]. Based on their structure, GameXPeptides were speculated to disrupt the insect immune response by interfering with the link between the innate and adaptive immune systems [58]. Rhabdopeptides, which were found in *X. stockiae* (bAST17.4) and *P. laumondii* subsp. *laumondii* (bALN19.5), have unknown activity, but it has structures similar to protease inhibitors. Therefore, this compound might degrade a range of proteins associated with immunity [59]. In addition, the hemocytes of waxworm treated with Rhabdopeptides showed severe loss of cytoskeletal structure, indicating programmed cell death [60].

The pathological change of digestive glands is used as a biomarker of induced toxicity in snails [61]. In the present study, we found hemocytes in the digestive cells. This result was also found in *B. glabrata* after exposure to *E. milii* latex, where hemocytes were present in the digestive gland, kidney, and mantle of the snail. The authors suggested that this phytochemical stimulated hemocyte proliferation (circulating and tissue specific) in the tissue, with hyalinocytes being most abundant, followed by granulocytes and blast cells. However, the hemocyte type does not influence the cellular immune response, but the number of hemocytes does [62].

The hermaphrodite glands in this study showed histopathological changes including a decrease of spermatozoa and the degeneration of oocytes. In addition, severe necrotic changes and degeneration of acini were also observed. The same results were observed by El-Khayat et al., who observed histopathological effects of plants extracts *A. arvensis* and *V. tinus* against *B. alexandrina.* The alterations in the hermaphrodite glands of the treated snails showed degeneration and necrotic changes in the acini [63]. The present results also agree with the studies on *B. glabrata* after exposure to *Eucalyptus camaldulensis* and niclosamide [64]. Also, when *B. alexandrina* and *B. truncates* were exposed to *P. canescens*, the hermaphrodite gland of these snails revealed that some ova organelles were deformed, and empty ova appeared. The sperm were distorted and reduced in number. Moreover, acinar epithelium showed necrotic changes in the form of partial destruction [65].

## Conclusions

Herein, we reported the first investigation of bacterial extracts to control *B. glabrata*, the intermediate host of *S. mansoni*. The results indicated that crude extracts of five bacteria showed good activity against *B. glabrata,* especially *P. laumondii* subsp. *laumondii* (bALN19.5_TH) and *X. stockiae* (bAST17.4_TH), which produced virulent secondary metabolites such as GameXPeptides and Rhabdopeptides. These extracts also caused histopathological alterations in the foot, digestive glands, and hermaphrodite glands of the snails. Therefore, these bacterial extracts might be effective molluscicides that could be used in the field to control the intermediate host snails.

## Supplementary Information

Additional file 1: Table S1 *P*-value of Bonferroni's post hoc for multiple comparisons based on comparison of the mortality of *Biomphalaria* *glabrata* between the bacterial extracts and negative control (DMSO) and positive control (niclosamide). A statistically significant difference was considered when a *P*-value showed less than 0.05.

Additional file 2: Table S2 The secondary metabolites derived from symbiotic bacteria *Xenorhabdus* and *Photorhabdus*. These metabolites were predicted from LC–MS/MS analysis of bacterial crude extracts.

Additional file 3 Figure S1 Network analysis of *Xenorhabdus* *stockiae* (bAST17.4_TH) showing 355 nodes connected in networks with at least one neighbor and a single node. Eighteen known natural product compounds were highlighted in the network. Figure S2 Network analysis of *Photorhabdus* *luminescens* (bAPY3.5_TH) showing 244 nodes connected in networks with at least one neighbor and a single node. Eighteen known natural product compounds were highlighted in the network.

## Data Availability

No datasets were generated or analyzed during the current study.

## Abbreviations

EPNs: Entomopathogenic nematodes
IJs: Infective juvenile stage
LB: Luria–Bertani
LC–MS/MS: Liquid chromatography–tandem mass spectrometry
MDA: Mass drug administration
MEGA: Molecular evolutionary genetics analysis
NBTA: Nutrient agar supplemented with bromothymol blue and triphenyl-2,3,5-tetrazolium chloride
PCR: Polymerase chain reaction
*recA*: Recombinase A
TSB: Tryptic soy broth
WHO: World Health Organization
