# Supplementary material for: Symbiotic bacteria associated with entomopathogenic nematodes showed molluscicidal activity against Biomphalaria glabrata, an intermediate host of Schistosoma mansoni
Source: Parasit Vectors. 2024 Dec 22;17:529. doi: 10.1186/s13071-024-06605-x (PMC11665110; doi:10.1186/s13071-024-06605-x)
Supplement: Supplementary file 2 — Additional file 2: Table S2 The secondary metabolites derived from symbiotic bacteria Xenorhabdus and Photorhabdus. These metabolites were predicted from LC–MS/MS analysis of bacterial crude extracts. [file 13071_2024_6605_MOESM2_ESM.docx]

**Table S1** The secondary metabolites derived from symbiotic bacteria *Xenorhabdus* and *Photorhabdus*. These metabolites were predicted from LC-MS/MS analysis of bacterial crude extracts.

| No. | *P. laumondii* subsp. *laumondii*  (bALN19.5_TH) | *X. stockiae*  (bAST17.4_TH) | *P. luminescens*  (bAPY3.5_TH) |
| --- | --- | --- | --- |
|  |  |  |  |
| 1. | Rhabdopeptide | Rhabdopeptide 1 or 2 | PE(18:1/0:0); [M+H]+ C23H47N1O7P1 |
| 2. | Versicoloritide A_22810 | Rhabdopeptide 5 | PE(16:1/0:0); [M+H]+ C21H43N1O7P1 |
| 3. | Xenofuranone | Rhabdopeptide 4 | prolylphenylalanine |
| 4. | L-Tryptophan | Rhabdopeptide M or N | GameXPeptide C |
| 5. | Photopyrone D | Rhabdopeptide | GameXPeptide A |
| 6. | GameXPeptide A | Rhabdopeptide O |  |
| 7. | Lumizinone B | Rhabdoplanin B |  |
| 8. | Spectral Match to PyroGlu-Ile-Lys from NIST14 | Xenobactin |  |
| 9. | val-leu-pro-val-pro | Rhabdopeptide_J |  |
| 10. | Spectral Match to Glu Phe from METLIN | Rhabdopeptide_I |  |
| 11. | prolylphenylalanine | Xeneprotide A |  |
| 12. | PE(18:1/0:0); [M+H]+ C23H47N1O7P1 | GameXPeptide A |  |
| 13. | PE(16:1/0:0); [M+H]+ C21H43N1O7P1 | GameXPeptide C |  |
| 14. |  | Pyrrolizixenamide A |  |
| 15. |  | Pyrrolizixenamide_B |  |
| 16. |  | Xenofuranone_A |  |
| 17. |  | Succinoadenosine |  |
| 18. |  | NCGC00381359-01_C14H18N2O3_Phenylalanine, prolyl- |  |
|  |  |  |  |
|  |  |  |  |
