# Supplementary material for: Symbiotic bacteria associated with entomopathogenic nematodes showed molluscicidal activity against Biomphalaria glabrata, an intermediate host of Schistosoma mansoni
Source: Parasit Vectors. 2024 Dec 22;17:529. doi: 10.1186/s13071-024-06605-x (PMC11665110; doi:10.1186/s13071-024-06605-x)
Supplement: Supplementary file 3 — Additional file 3 Figure S1 Network analysis of Xenorhabdus stockiae (bAST17.4_TH) showing 355 nodes connected in networks with at least one neighbor and a single node. Eighteen known natural product compounds were highlighted in the network. Figure S2 Network analysis of Photorhabdus luminescens (bAPY3.5_TH) showing 244 nodes connected in networks with at least one neighbor and a single node. Eighteen known natural product compounds were highlighted in the network. [file 13071_2024_6605_MOESM3_ESM.docx]

**Figure S1** Network analysis of *Xenorhabdus stockiae* (bAST 17.4_TH) showing 355 nodes connected in networks with at least one neighbor and a single node. Eighteen known natural product compounds were highlighted in the network.

**Figure S2** Network analysis of *Photorhabdus luminescens* (bAPY 3.5_TH) showing 244 nodes connected in networks with at least one neighbor and a single node. Eighteen known natural product compounds were highlighted in the network.
